# Same-session endoscopic retrograde direct cholangioscopy-assisted laparoscopic cholecystectomy: real-time intraluminal navigation for prevention of cystic duct stone residual

**DOI:** 10.1055/a-2897-7560

**Published:** 2026-06-30

**Authors:** Shan-Shan Hu, Wei-Hui Liu

**Affiliations:** 1 Department of Gastroenterology and HepatologySichuan Provincial People’s Hospital, School of Medicine, University of Electronic Science and Technology of ChinaChengduSichuan ProvinceChina

**Fig. 1:**
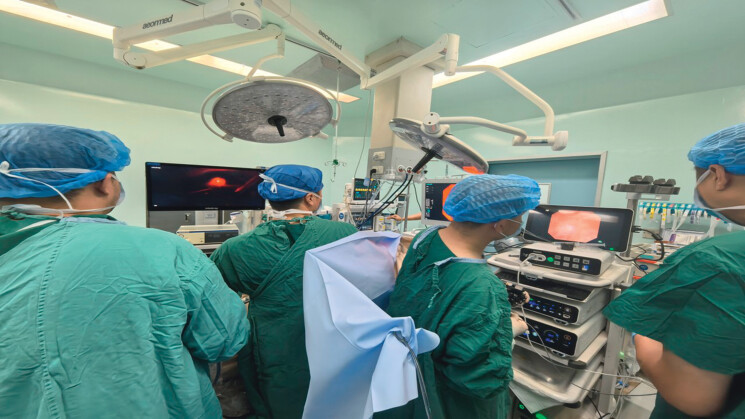
Same-session interdisciplinary operative setting.

Previous reports have positioned endoscopic retrograde direct cholangioscopy (ERDC) as a primary therapeutic modality for complex biliary diseases such as Mirizzi syndrome. 1​ We report a fundamentally distinct application: ERDC deployed as a dedicated real-time surgical navigational platform during routine laparoscopic cholecystectomy (LC) in a same-session, interdisciplinary operative setting ( Fig. 1 ). This paradigm shift addresses the 5% to 12% cystic duct stone residual rate 2​ ​3​ by providing direct intraluminal visualization for extraluminal dissection guidance and definitive stone clearance confirmation, while eliminating the radiation exposure inherent to conventional intraoperative cholangiography. 4​

**Fig. 2:**
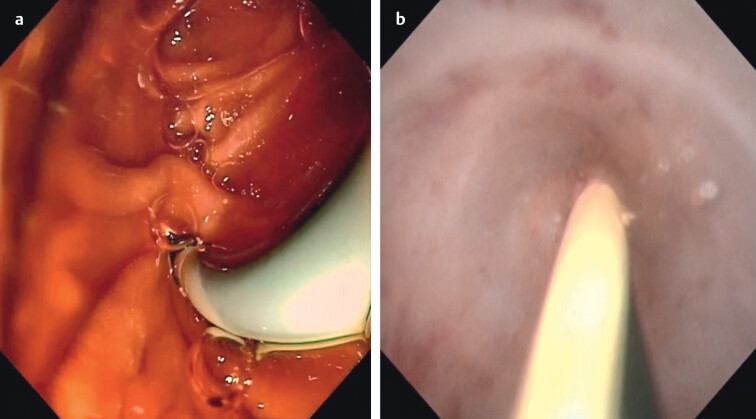
Direct cholangioscopic view of CBD entry via transpapillary route.

A 68-year-old man with recurrent biliary colic and ambiguous cystic duct anatomy on magnetic resonance cholangiopancreatography underwent same-session ERDC-assisted LC. In the same operative suite, the endoscopy and surgical teams operated concurrently ( Fig. 1 ). Under ERDC, the endoscopist precisely navigated into the common bile duct (CBD), then selectively intubated the cystic duct, identified stone location, and demarcated the cystic duct take-off angle ( **Figs** 2 **,** 3 ). The laparoscopic team leveraged this real-time intraluminal landmark — visible simultaneously on the endoscopy monitor within the operative suite — to safely dissect a severely fibrotic Calot triangle, eliminating speculative anatomical interpretation and fluoroscopy dependence ( Fig. 4 ). After cystic duct transection, ERDC reconfirmed complete stone clearance within the residual cystic duct stump through direct intraluminal inspection ( Fig. 5 ).

**Fig. 3:**
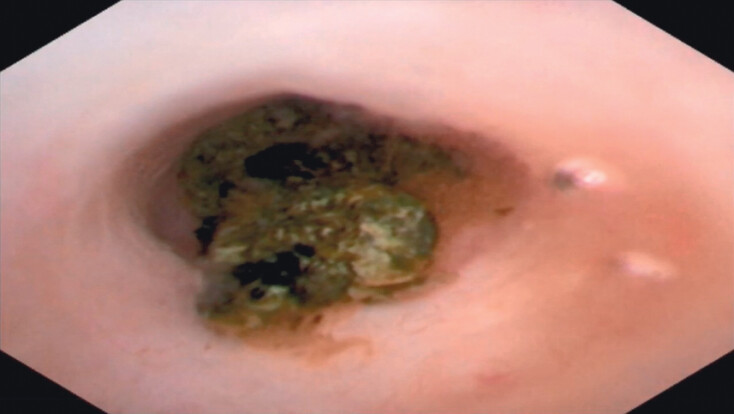
Selective cystic duct intubation with direct visualization of intraluminal stone.

**Fig. 4:**
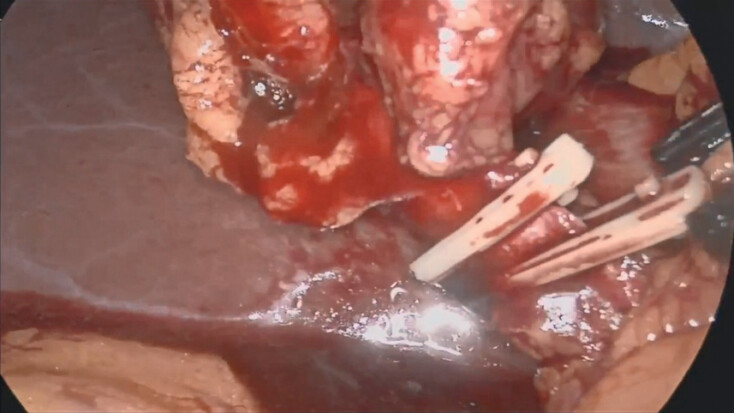
The laparoscopic team dissected the fibrotic Calot triangle under real-time ERDC guidance.

**Fig. 5:**
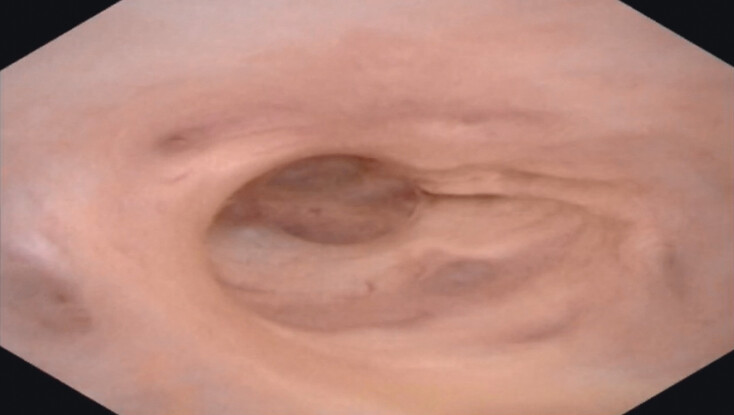
Post-transection ERDC confirmation of complete stone clearance.

This video ( Video 1 ) demonstrates the paradigm shift of ERDC from a therapeutic tool to a surgical navigation platform in a same-session collaborative model. By directly providing visual evidence for anatomical confirmation and stone clearance in the same operative session, ERDC overcomes the fundamental limitation of conventional cholangiography in preventing cystic duct stone residual, offering significant clinical scalability.

**Video 1** Same-session endoscopic retrograde direct cholangioscopy-assisted laparoscopic cholecystectomy: real-time intraluminal navigation for prevention of cystic duct stone residual.


Endoscopy_UCTN_Code_TTT_1AR_2AH
